# Supplementary material for: Molecular crowding effects on protein stability in a bacterial proteome
Source: Sci Rep. 2026 Jan 21;16:5908. doi: 10.1038/s41598-026-35990-9 (PMC12894976; doi:10.1038/s41598-026-35990-9)
Supplement: Supplementary file 2 — Supplementary Material 2 [file 41598_2026_35990_MOESM2_ESM.zip › AllTheTPPData/NvF400/Panobinostat_Vignette/QCplots.pdf]

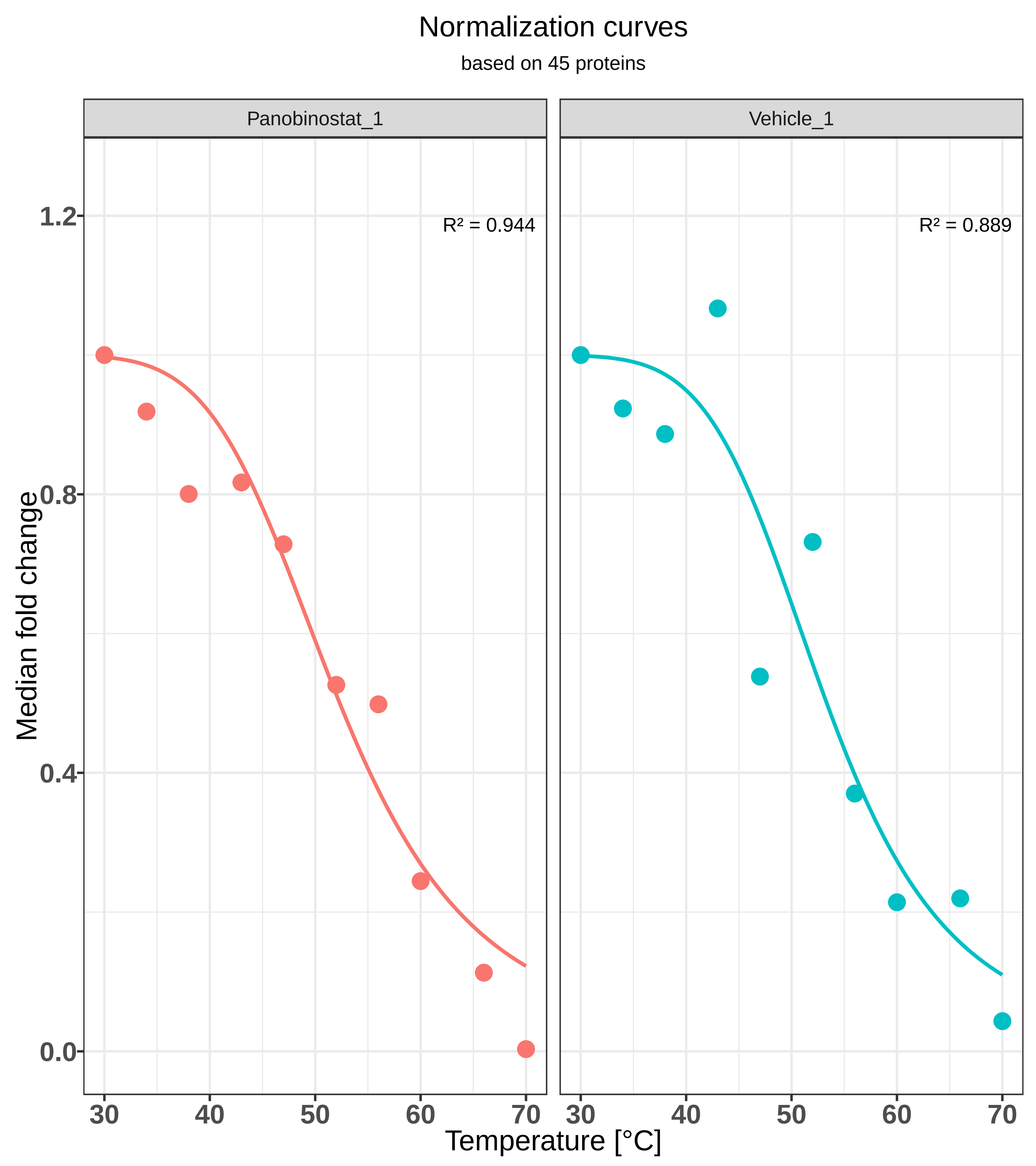

Normalization curves
based on 45 proteins
Panobinostat_1
Vehicle_1
R² = 0.944
R² = 0.889
Median fold change
1.2
0.8
0.4
0.0
30
40
50
60
70
Temperature [°C]

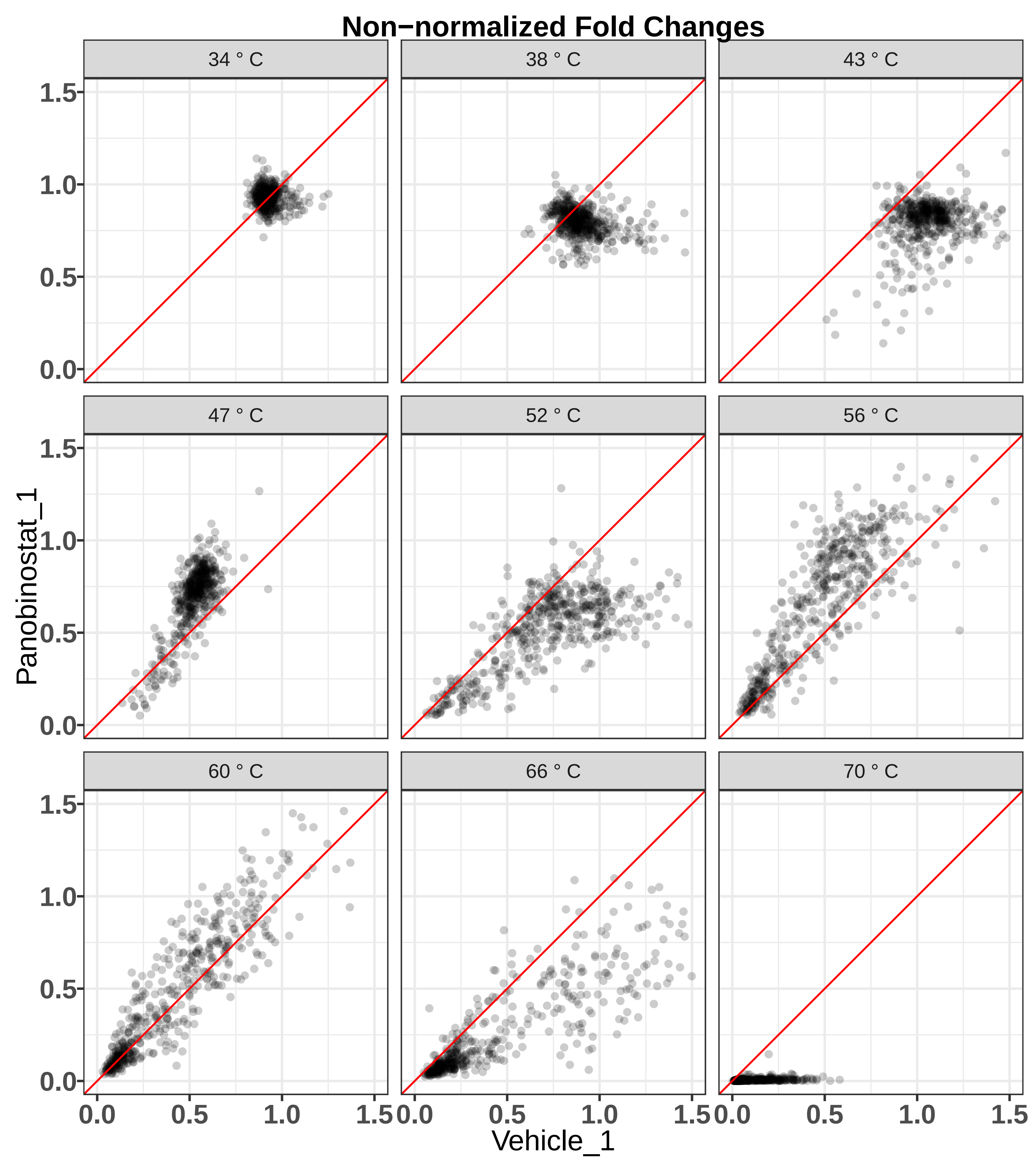

Non−normalized Fold Changes
34 ° C
38 ° C
43 ° C
47 ° C
52 ° C
56 ° C
60 ° C
66 ° C
70 ° C
Panobinostat_1
Vehicle_1
0.0
0.5
1.0
1.5

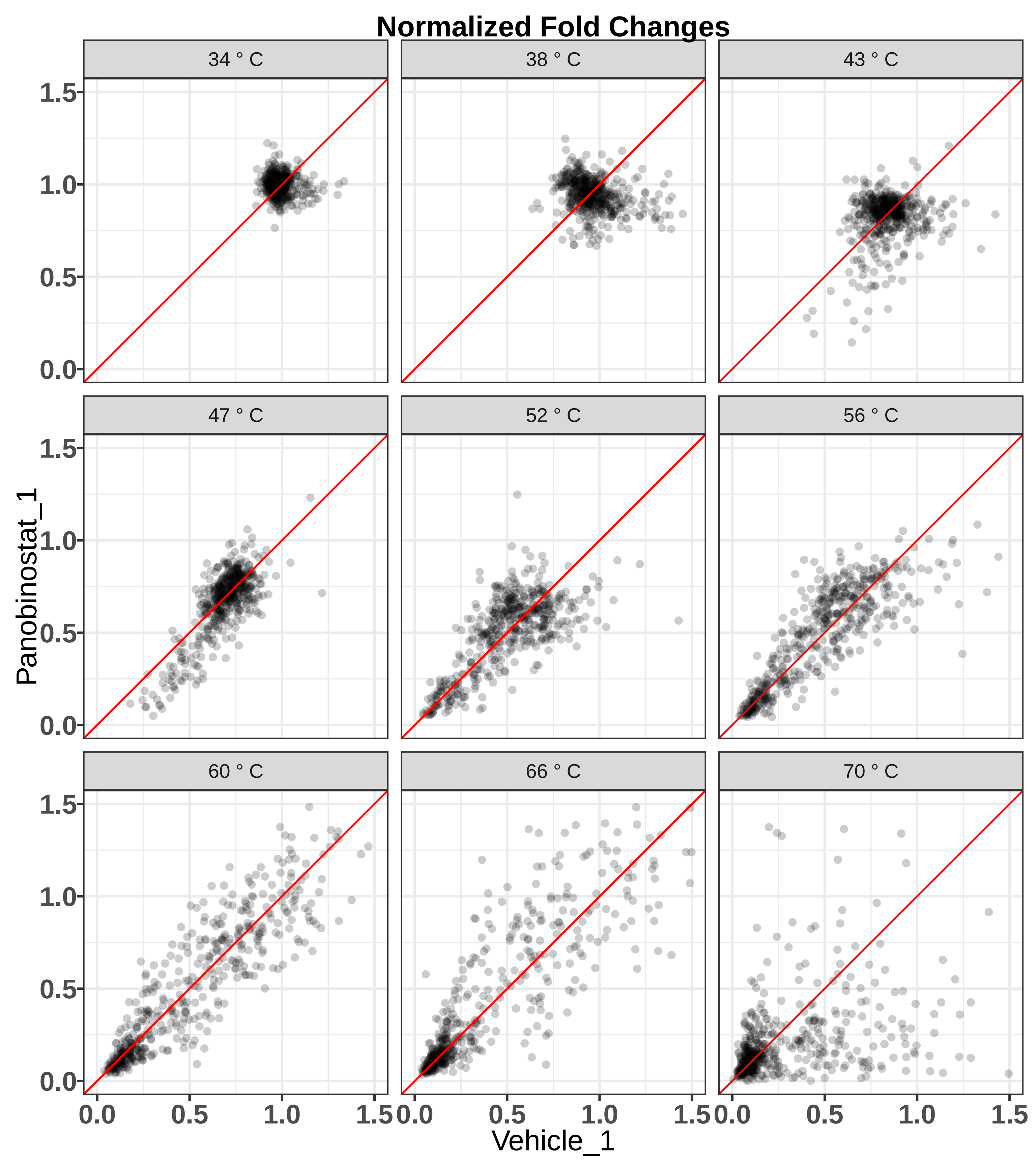

Normalized Fold Changes
34 ° C
38 ° C
43 ° C
47 ° C
52 ° C
56 ° C
60 ° C
66 ° C
70 ° C
Panobinostat_1
Vehicle_1
0.0
0.5
1.0
1.5

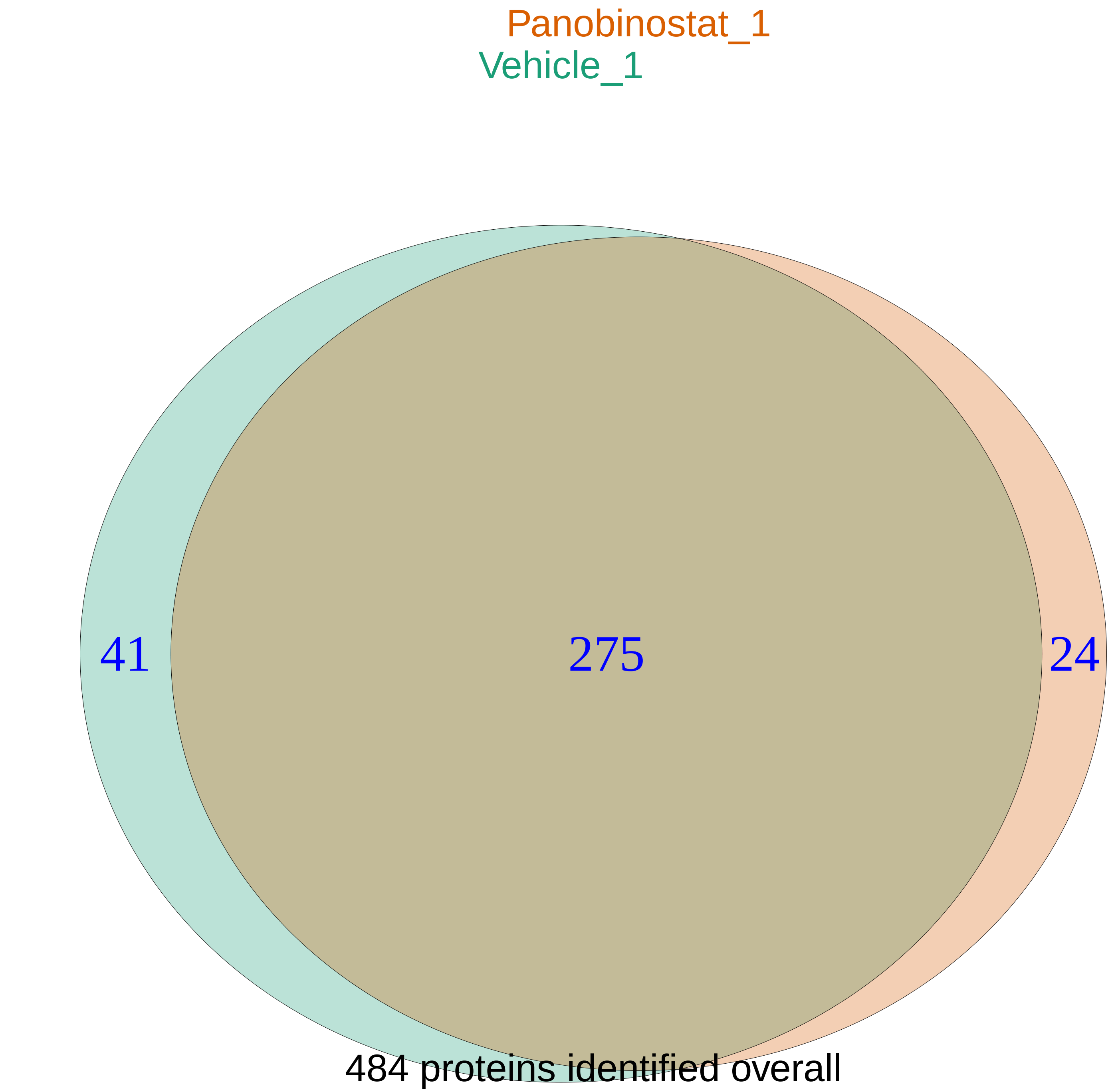

484 proteins identified overall

	R2 >= 0.8	R2 < 0.8	Total
Vehicle_1	[illegible]	[illegible]	[illegible]
Panobinostat_1	299	185	484

Panobinostat_1_vs_Vehicle_1

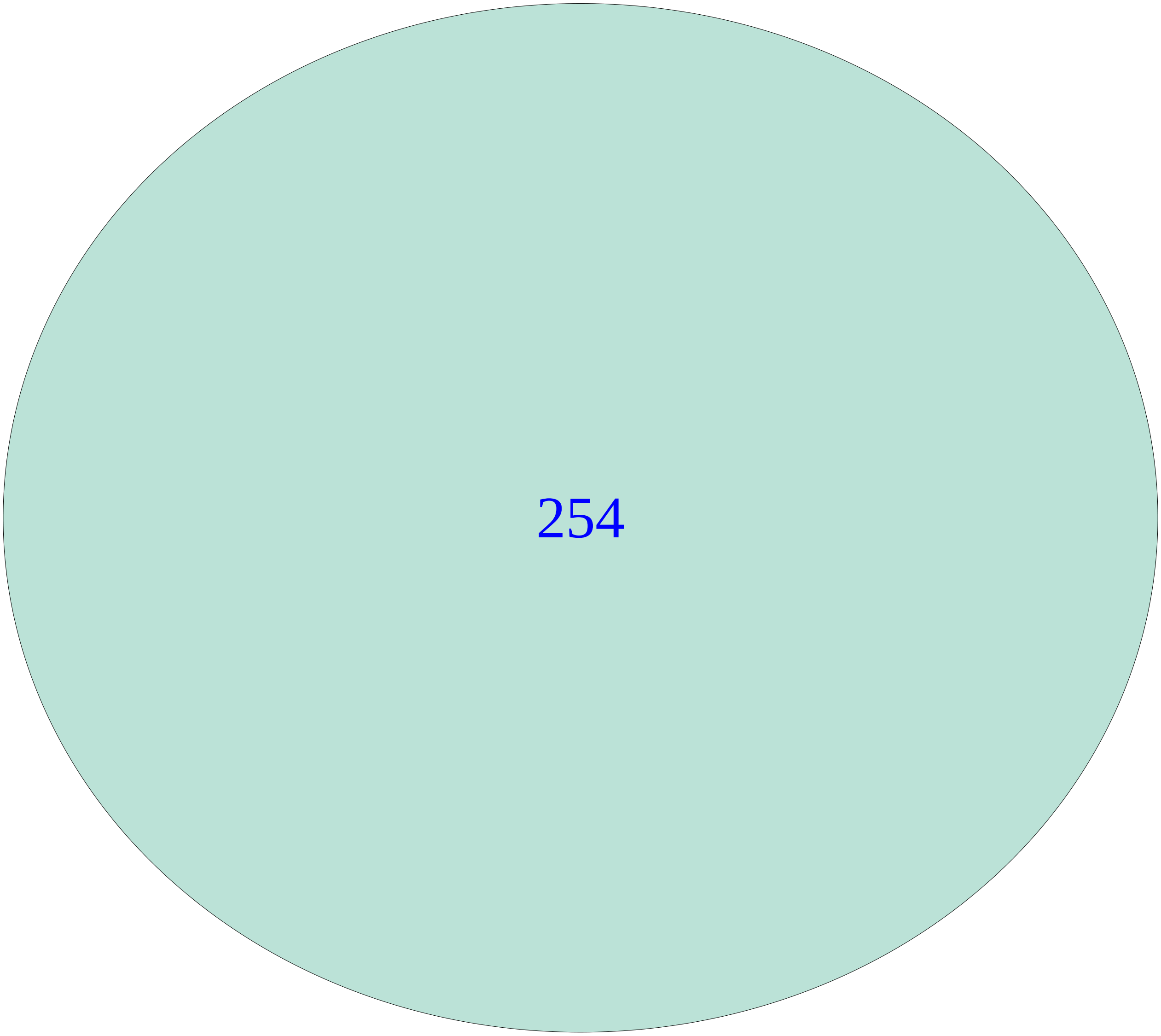

	passed_filter	filtered_out	Total
nobinostat_1_vs_Vehicle_1	254	230	484

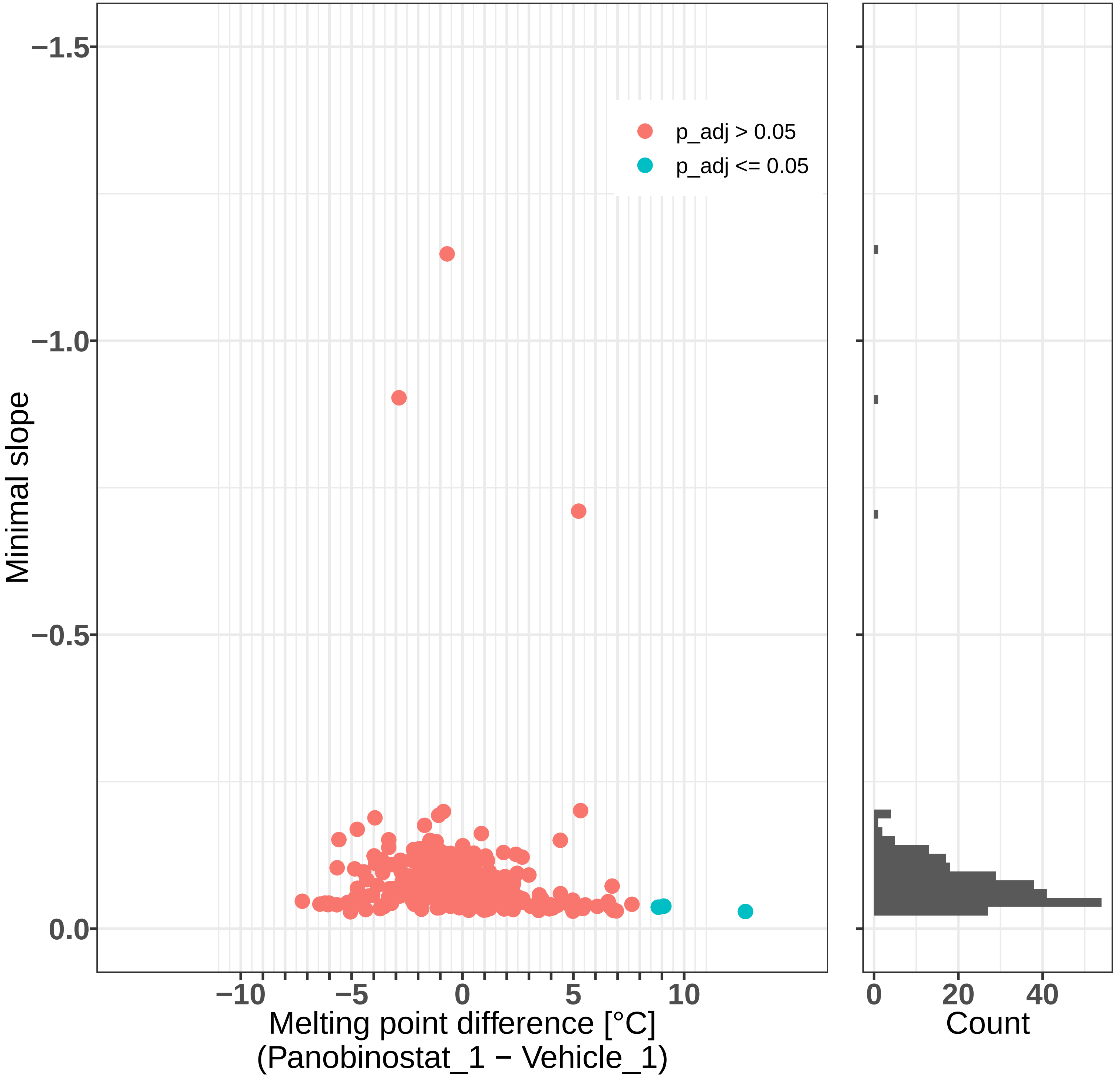

n = 252

Panobinostat_1 vs. Vehicle_1

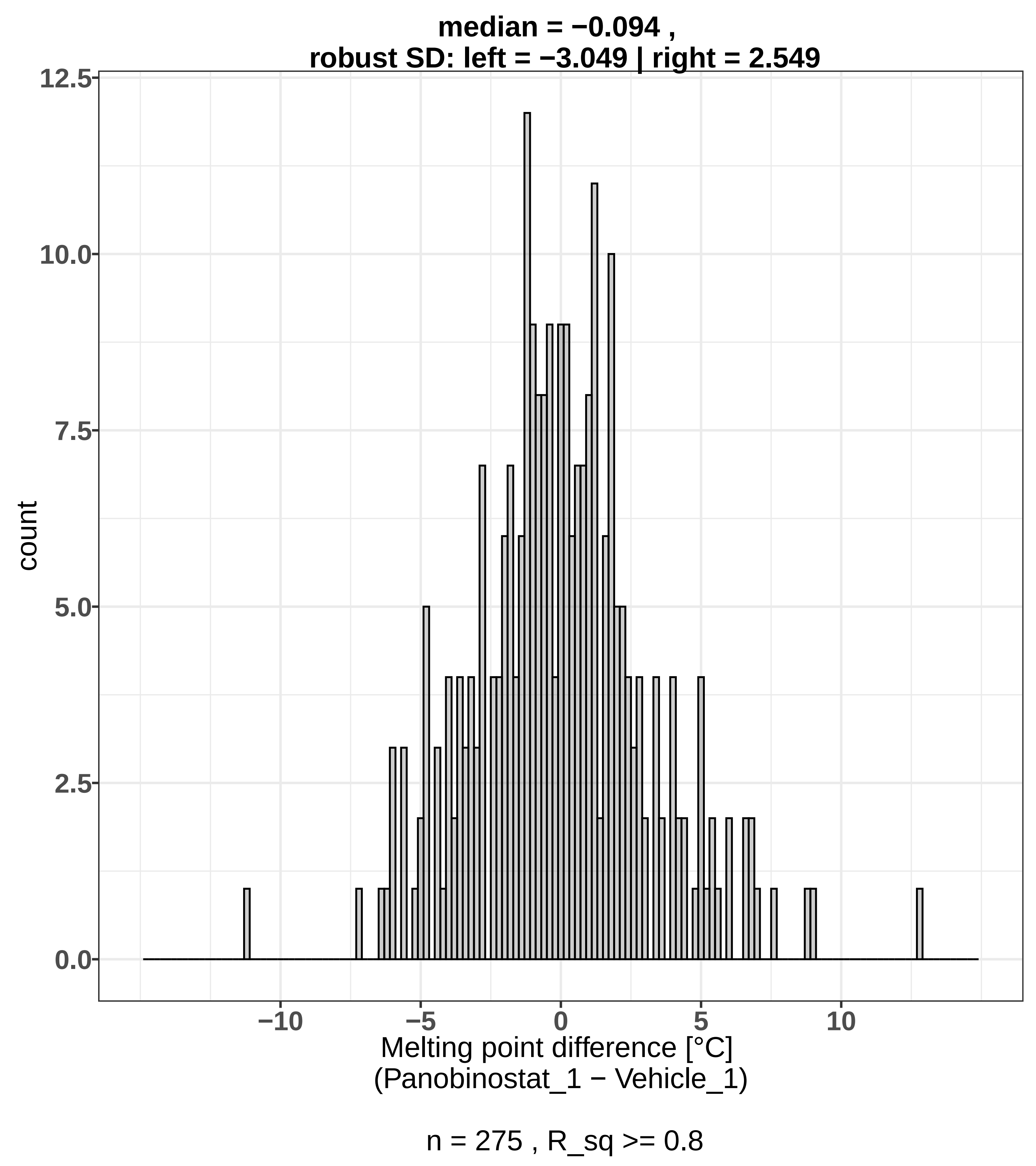
median = −0.094 ,
robust SD: left = −3.049 | right = 2.549
12.5
10.0
7.5
5.0
2.5
0.0
count
−10
−5
0
5
10
Melting point difference [°C]
(Panobinostat_1 − Vehicle_1)
n = 275 , R_sq >= 0.8

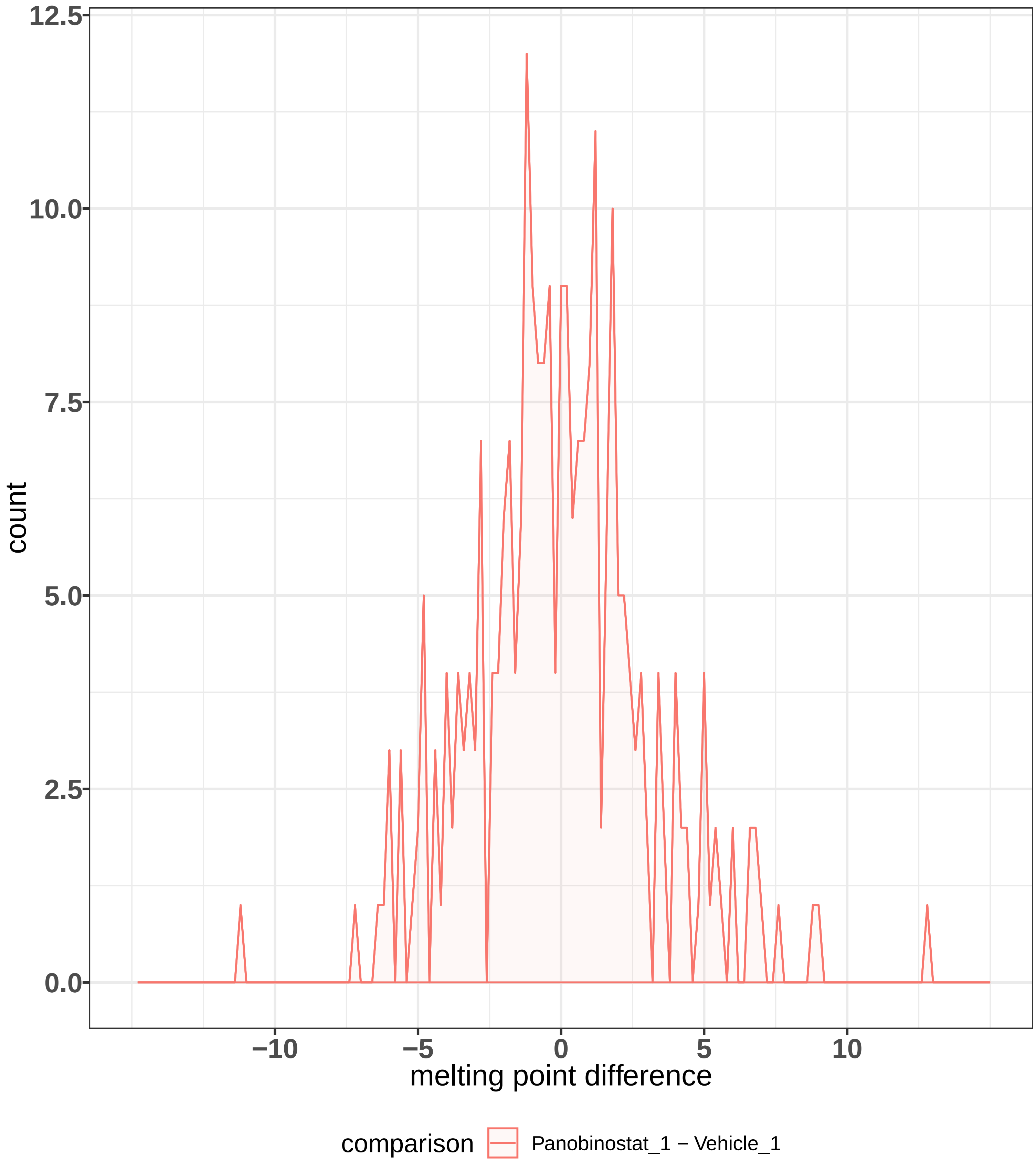

12.5
10.0
7.5
5.0
2.5
0.0
count
−10
−5
0
5
10
melting point difference
comparison
Panobinostat_1 − Vehicle_1
